# Commodity Thermoplastic Elastomer-Enabled Templated Synthesis of Large-Pore Ordered Mesoporous Materials

**DOI:** 10.1021/acsomega.5c00553

**Published:** 2025-03-12

**Authors:** Anthony Griffin, Parker Frame, Yizhi Xiang, Zhe Qiang

**Affiliations:** † School of Polymer Science and Engineering, University of Southern Mississippi, Hattiesburg, Mississippi 39406, United States; ‡ Department of Chemical and Biomedical Engineering, University of Missouri, Columbia, Missouri 65211, United States

## Abstract

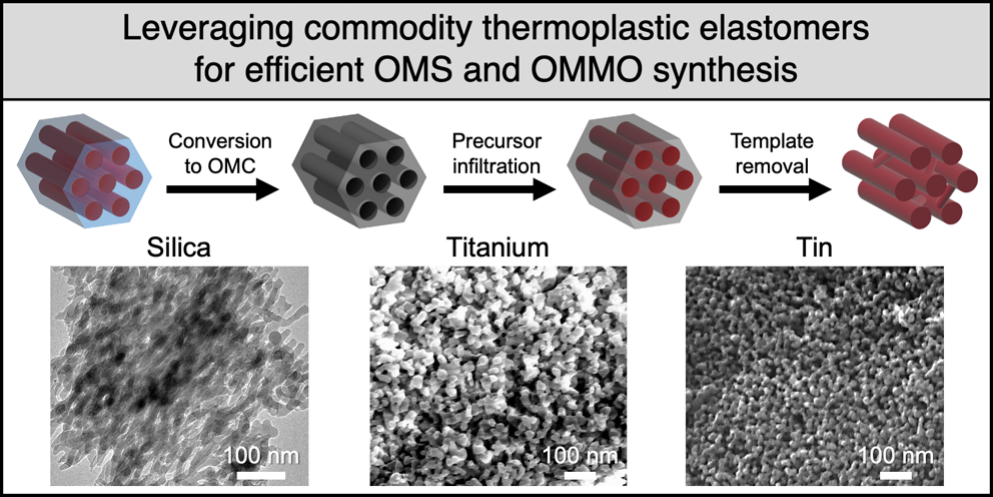

Fabrication of ordered mesoporous materials (OMMs) has predominantly relied on templating-based methods. However, these methods are constrained by several limitations, especially the limited pore sizes attainable with commercially available surfactants used as structure-directing agents. To unlock the full potential of the OMMs, it is essential to develop synthetic strategies that facilitate the production of large-pore OMMs using scalable processes and cost-effective precursors. This work demonstrates the use of thermoplastic elastomer (TPE)-derived carbon replicas for synthesizing ordered mesoporous silica (OMS) and metal oxides (OMMOs) via precursor infiltration and template removal. The nanostructural evolution of the resulting inorganic materials was systematically investigated. Specifically, using tetraethyl orthosilicate (TEOS) as a silica precursor, this method can produce an OMS with relatively large pores. To establish the generalizability of this process, the fabrication approach was extended to other commercially available TPEs with varied chemical compositions and molecular weights while consistently resulting in ordered structures. Additionally, this synthetic strategy can be successfully applied to the production of OMMOs, including tin and titanium oxide matrix chemistries, yielding pore sizes of 16.0 and 19.2 nm, respectively. By developing a general method and using low-cost precursors, this work presents a scalable approach for fabricating large-pore OMMs with tunable pore textures and matrix chemistries.

## 1 Introduction

Ordered mesoporous materials (OMMs), containing pore sizes between 2 and 50 nm, hold great interest within the broader scientific community due to their unique combination of uniform pore channels and high surface area,^1^ which can be applied to various applications, including water remediation,^2−4^ catalysis,^5−7^ energy storage,^8−10^ and drug delivery.^11−13^ Particularly, significant research efforts have been dedicated to developing synthetic strategies for the synthesis of OMMs with controlled pore textures to expand their utility and potential.^14^ In general, most OMMs are prepared via templating-based methods, including using supramolecular aggregates via coassembly (soft-templating), prefabricated mesoporous solids (hard-templating), or both.^15−17^ While effective for fabricating the OMMs, these methods can be limited in achieving large pore sizes by using commercially available templating agents. This is because most surfactant templates have a molecular weight below 5000 g/mol, and as a result, their derived micelle size is often less than 10 nm. Many works have indicated that small mesopore sizes may result in pore blockage and sluggish transport of guest molecules in various applications,^18−22^ further highlighting the need for preparing large-pore OMMs.

It has been recognized that the development of large mesopores in OMM can greatly improve their performance in many applications, via enhanced transport and sorption of large-size guest molecules.^23−25^ For example, Wu et al. investigated the impact of ordered mesoporous carbon (OMC) pore size on sodium-ion battery performance and found that larger pore size (∼6.5 nm) resulted in higher sodiation capacity and improved long-term cycle stability compared to their counterparts containing smaller mesopores (4.7 nm).^26^ Large-mesopore OMM synthesis can be achieved by several approaches, such as through the inclusion of pore expanders and using block copolymer templates with high molecular weight. For instance, Kruk et al. demonstrated the synthesis of ordered mesoporous silica (OMS) with large pore diameters (∼20 nm) through the combination of a Pluronic surfactant (F127, EO_106_PO_70_EO_106_) and a swelling agent (toluene), which partially solubilizes the surfactant micelle and acts as a micelle expander.^16^ Furthermore, tailored block copolyethers, or “reverse Pluronics” (PPO_*n*/2_-PEO_*m*_-PPO_*n*/2_) with defined molecular weights, can be prepared through organocatalytic polymerization and utilized as templates for OMCs, which can result in pore diameters ranging from 6 to 18 nm.^27^ Additionally, Watkins et al. synthesized large-pore OMC films (∼40 nm) using bottlebrush block copolymers, with molecular weight up to 500 000 g/mol, through a soft-templating approach followed by rapid thermal annealing.^18^ While tailor-made block copolymers and pore expanders can achieve larger mesopore sizes, they often involve high cost and/or sophisticated polymer synthesis. Enabling scaled production of OMMs requires the utilization of low-cost precursors and simple processes.

Herein, this work demonstrates a generalized method to prepare OMMs by leveraging commodity, low-cost thermoplastic elastomers (TPEs) as structure-directing agents, in combination with a hard-templating method. Polystyrene-*block*-poly(ethylene-*ran*-butylene)-*block*-polystyrene (SEBS) can be directly transformed into ordered mesoporous carbons, serving as templates for synthesizing ordered mesoporous silica (OMS) and metal oxides (OMMOs). The evolution of the nanostructure and chemical composition was systematically studied during precursor infiltration and template decomposition. OMS was successfully prepared by infiltrating precursors into the carbon template, followed by calcination for template removal, achieving a relative silica content of up to 17.6 wt % and an average pore size of 8.6 nm. The versatility of this process was demonstrated by using other commercial TPEs with varied molecular weights and compositions. Our method was further extended to fabricate OMMOs, including tin oxide and titanium oxide, which can yield ordered nanostructures with average pore sizes of 16.0 and 19.2 nm, respectively.

## 2 Experimental Section

### 2.1 Materials

SEBS [*M*_n_: 89 000 g/mol, ϕ_PS_ = 0.20, *Đ*: 1.56 and 118 000 g/mol, ϕ_PS_ = 0.20, *Đ*: 1.59], polystyrene-*block*-polybutadiene-*block*-polystyrene (SBS) [*M*_n_: 140 000 g/mol, ϕ_PS_ = 0.33, *Đ*: 1.15], tetraethyl orthosilicate (TEOS; > 98%), titanium(IV) chloride (99.9%), tin(IV) chloride (98%), ethanol (>99.5%), and hydrochloric acid (37%) were purchased from Sigma-Aldrich. A Millipore Sigma Milli-Q IQ 7003 ultrapure lab water purification system was used to obtain deionized (DI) water.

### 2.2 Preparation of Ordered Mesoporous Carbon (OMC) Template

In a typical synthesis of the OMC, 0.5 g of TPE was introduced into a 100-mL round-bottomed flask containing 5 g of sulfuric acid and a stir bar. The mixture was then placed in a thermal bead bath preheated to 150 °C and reacted for 4 h. Following the completion of the reaction, the mixture was allowed to cool to room temperature and passed through a glass fritted funnel. Residual byproducts and acid were then removed through three cycles of washing with 150 mL of DI water. The cross-linked polymer was dried at 45 °C overnight and then heated in a tube furnace (MTI Corporation OTF-1200×) under a nitrogen environment up to 600 °C at a rate of 1 °C/min, followed by heating to 800 °C at a rate of 5 °C/min.

### 2.3 Synthesis of OMS and OMMOs

To fabricate the OMS, 0.637 g of TEOS, 0.3 g of prepared OMC, and 15 g of 1.6 M HCl were placed in a 50-mL round-bottom flask with a stir bar. The reaction flask was then heated to 45 °C for varying amounts of time. The product was then separated with a glass fritted funnel, dried at room temperature overnight, and heated under a nitrogen environment up to 600 °C at a rate of 1 °C/min, followed by heating to 800 °C at a rate of 5 °C/min. Subsequently, the sample was then heated to 550 °C for 3 h in air with a ramp rate of 1 °C/min, which thermally decomposed carbon and yielded pure silica materials. To fabricate OMMOs, 0.45 g of metal precursor and 0.3 g of prepared OMC were added to 5 mL of an ethanol/DI water mixture (70:30 w/w). The mixture was then stirred for 24 h, and the product was separated with a glass fritted funnel, dried overnight, and heated under a nitrogen environment up to 600 °C at a rate of 1 °C/min, followed by heating to 800 °C at a rate of 5 °C/min. Subsequently, to remove the carbon template, the sample was heated to 750 °C for 3 h in air at a ramp rate of 1 °C/min.

### 2.4 Characterization

A Tristar II 3020 (Micromeritics) was used for nitrogen physisorption measurements, with pore size distributions determined with nonlocal density functional theory (NLDFT) and surface areas by Brunauer–Emmett–Teller (BET) analysis. Small-angle X-ray scattering (SAXS) measurements were performed at the 11-BM Complex Materials Scattering beamline at the National Synchrotron Light Source II within the Brookhaven National Laboratory, with an X-ray energy of 12 keV and a sample-to-detector distance of 5.05 m. Domain spacings were determined by the equation *d* = 2π/q*. A Zeiss Ultra 60 field-emission scanning electron microscope (SEM) with an accelerating voltage of 14 kV was used to further probe the morphology of the mesoporous materials. Thermogravimetric analysis (TGA) measurements were carried out with a TA Instruments Discovery Series 550 under air with a ramp rate of 10°/min up to 800°. A Rigaku MiniFlex X-ray diffractometer (XRD) was used for XRD measurements with Cu Kα radiation in the 2θ range of 10°–90° with a scan speed of 1°/min. Data reduction and analysis were carried out with SmartLab Studio II software. A Thermo Fisher ESCALAB Xi+ spectrometer was used for X-ray photoelectron spectroscopy (XPS) analysis with a monochromatic Al X-ray source (1486.6 eV) and a MAGCIS Ar+/Arn+ gas cluster ion sputter gun. The analysis chamber had a base pressure of 3 × 10^–7^ mbar, and a takeoff angle of 90° from the surface was used during data collection. Spectral analysis was carried out with the Thermo Fisher Avantage software. A JEOL JEM-1400 Electron Microscope equipped with a Gatan Rio 9 CMOS camera was used to capture transmission electron microscopy (TEM) images at an acceleration voltage of 120 kV.

## 3 Results and Discussion

**Figure 1:**
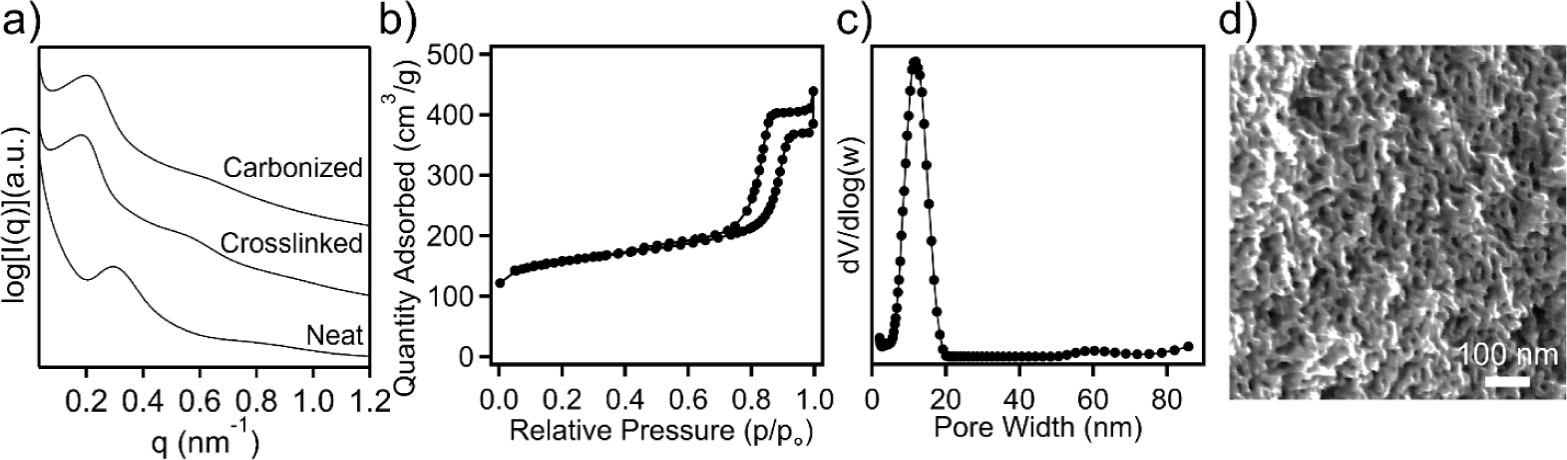
(a) SAXS profiles for neat, cross-linked, and carbonized SEBS89. (b) Nitrogen sorption isotherm, (c) pore size distribution, and (d) SEM micrograph of SEBS89-derived OMC.

Thermoplastic elastomers (TPEs) were first converted to OMCs through a two-step process of sulfonation-induced cross-linking and pyrolysis.^28^ In this study, a commercial SEBS was first used as a model system with a number-averaged molecular weight (*M*_n_) of 89 000 g/mol, a polydispersity index (*Đ*) of 1.56, and a polystyrene (PS) volume fraction of 0.20 (SEBS89). Specifically, SEBS was exposed to concentrated sulfuric acid at 150 °C for 4 h, which results in selective cross-linking of the majority polyolefin block, whereas the minority polystyrene blocks are sulfonated but unable to undergo cross-linking. In the poly(ethylene-*ran*-butylene) (PEB) phase, sulfonic acid groups can homolytically dissociate to form double bonds throughout the polymeric backbone, enabling further reactions of intermolecular radical–radical couplings and cross-linking.^29,30^ The impact of the sulfonation reaction on the SEBS nanostructure was probed through SAXS, where domain spacing expanded significantly from 22.5 to 35.3 nm following cross-linking (Figure 1a). The increased domain spacing following the cross-linking reaction can be attributed to the swelling of the PS and PEB phase as sulfonic acid groups are incorporated.^31^ Upon pyrolysis at 800 °C, the majority PEB phase is converted to a carbon matrix, while the minority PS blocks decompose, forming an ordered mesoporous morphology. Retention of an ordered nanostructure following carbonization was observed in the presence of a primary ordering peak (q*), which corresponds to a domain spacing of 31.8 nm. It is worth noting that pore textures can be modulated through varying precursor properties, such as the TPE’s molecular weight and the PS volume fraction, as well as process conditions, including cross-linking reaction temperature and time, as well as carbonization temperature.^28,31^ To further assess the pore textures of synthesized OMCs, nitrogen physisorption experiments were carried out where a type IV isotherm was observed, which is characteristic of mesoporous materials (Figure 1b). The SEBS89-derived OMC from direct pyrolysis has a BET surface area of 442 m^2^/g and a pore volume of 0.47 cm^3^/g. Figure 1c shows the pore size distribution, with a monomodal distribution centered at 11.8 nm. Furthermore, uniform pore sizes can be further confirmed through SEM imaging in Figure 1d with an average pore size of 11.9 nm.

**Figure 2:**
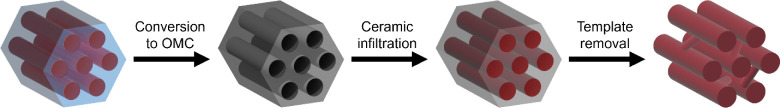
A simplified illustration demonstrating the conversion of TPEs to OMCs and their use as templates for the preparation of ordered mesoporous ceramics.

A generalized schematic illustration of the fabrication of OMMs from TPE templates is provided in Figure 2, including the synthesis of TPE-derived OMCs followed by inorganic precursor infiltration, resulting in carbon–silica nanocomposites. Subsequently, nanocomposites can be converted to OMMs through the decomposition of the carbon template.

**Figure 3:**
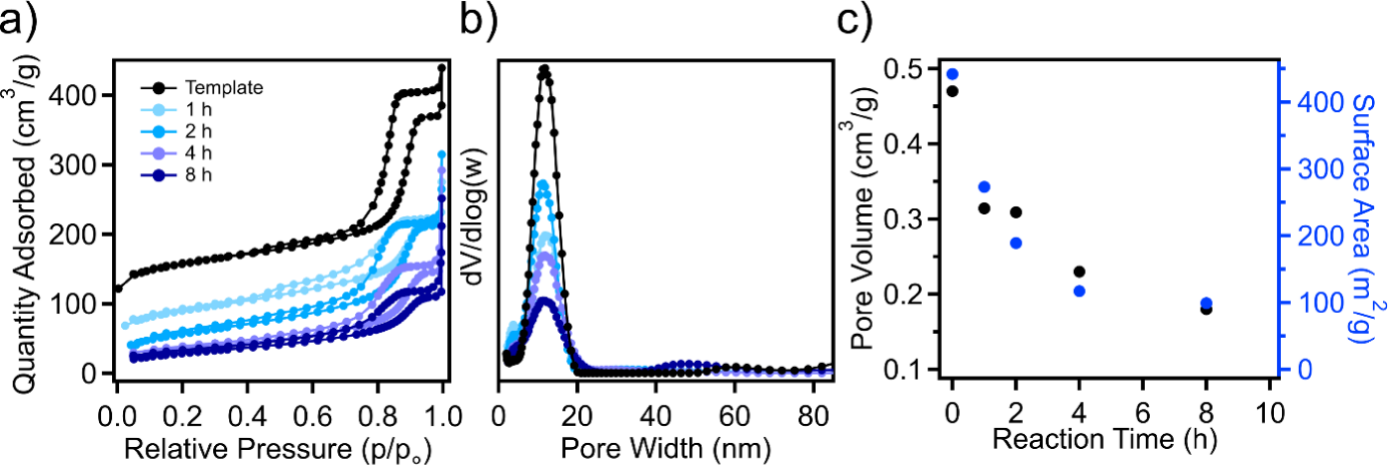
(a) Nitrogen sorption isotherms, (b) pore size distributions, and (c) pore volume and surface area as a function of the reaction time for TEOS-loaded OMCs.

To assess how incorporating silica impacts the resulting carbon–silica composite’s pore textures, nitrogen physisorption isotherms are shown in Figure 3a as a function of reaction time. Increased reaction time for TEOS infiltration and hydrolysis is observed to significantly alter the type IV isotherm, while the corresponding pore size distributions also exhibit a reduced intensity (Figure 3b), although the averaged pore size remains generally unaffected. The impact on pore volume and surface area can be observed in Figure 3c, where the surface area decreased from 442 m^2^/g in the template to 273 m^2^/g following 1 h of reaction and gradually declined with increasing reaction time (189 m^2^/g, 117 m^2^/g, and 99 m^2^/g after 2, 4, and 8 h, respectively). The pore volume displayed a similar behavior, reducing from 0.47 cm^3^/g in the template to 0.31 cm^3^/g, 0.23 cm^3^/g, and 0.18 cm^3^/g following 1, 2, and 8 h of reaction time, respectively. The decrease in both surface area and pore volume at low infiltration times suggests that TEOS is infiltrating within the mesoporous channels of the carbon template, as is expected in the hard templating method. Figure S1a,b shows the nitrogen physisorption isotherm and pore size distribution for the composites following various reaction times, where increased reaction time results in broadening of the pore size distribution. The pore size distribution remains relatively centered at 11.7 nm across reaction times, while the carbon template exhibited an averaged pore size of 11.9 nm. Figure S2 shows how the surface area and pore volume of the composites shift with varied reaction times. Interestingly, the surface area and pore volume increased significantly following the pyrolysis step and are relatively consistent across all reaction times, ∼440 m^2^/g and ∼0.47 cm^3^/g. The increased surface area and porosity could be due to physical activation of the carbon framework from the generation of water and other byproducts during the pyrolysis of TEOS.

**Figure 4:**
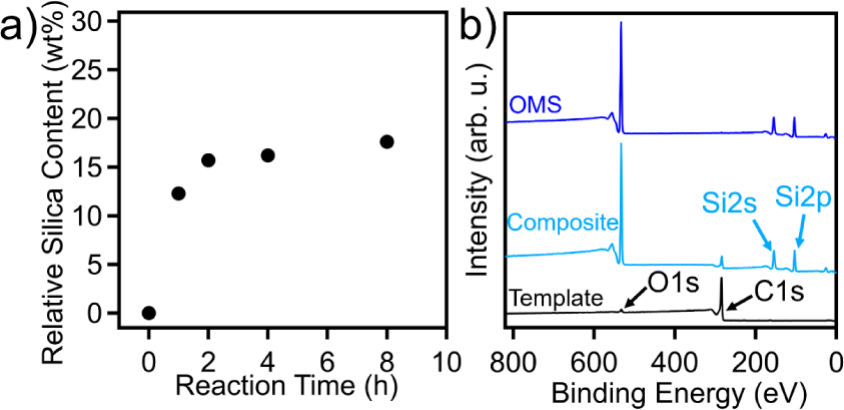
(a) Relative silica content as a function of the reaction time for SEBS89-derived OMS. (b) XPS survey scans for the SEBS89-derived carbon template, carbon–silica composite following 8 h of reaction, and silica following template decomposition.

Following the formation of carbon–silica composites, samples were then exposed to 550 °C under air to remove the carbon template and form the OMS products. Relative silica content was determined through TGA experiments (Figure S3) and is depicted in Figure 4a. A relative silica content of 12.2 wt % was found after 1 h of reaction, which increased slightly to 15.6 wt % after 2 h and up to 17.6 wt % after 8 h, indicating the majority of silica infiltration occurs rapidly. The elemental composition of the resulting silica can be determined through XPS survey scans (Figure 4b), where the carbon template was found to contain ∼3.3 at% oxygen and ∼0.7 at% sulfur prior to TEOS infiltration. The binding environments of heteroatoms in the carbon framework revealed from high-resolution XPS scans can be found in Figure S4. In comparison, the carbon–silica composite, prepared from an 8 h infiltration time, had an elemental composition of ∼57.8 at% carbon, ∼27.3 at% silica, and ∼14.9 at% oxygen. Figure S5 shows the high-resolution XPS scans of C 1*s*, Si 2*p*, and O 1*s*, respectively. Specifically, Si 2*p* has a single peak at 103.6 eV and O 1*s* has a peak at 532.9 eV, both suggesting the formation of SiO_2_. After decomposition of the carbon template under air, no trace of carbon was observed and a 1:2 atomic ratio of Si and O was found, indicating only pure SiO_2_ was left, as evidenced by high-resolution XPS scans in Figure S6.

**Figure 5:**
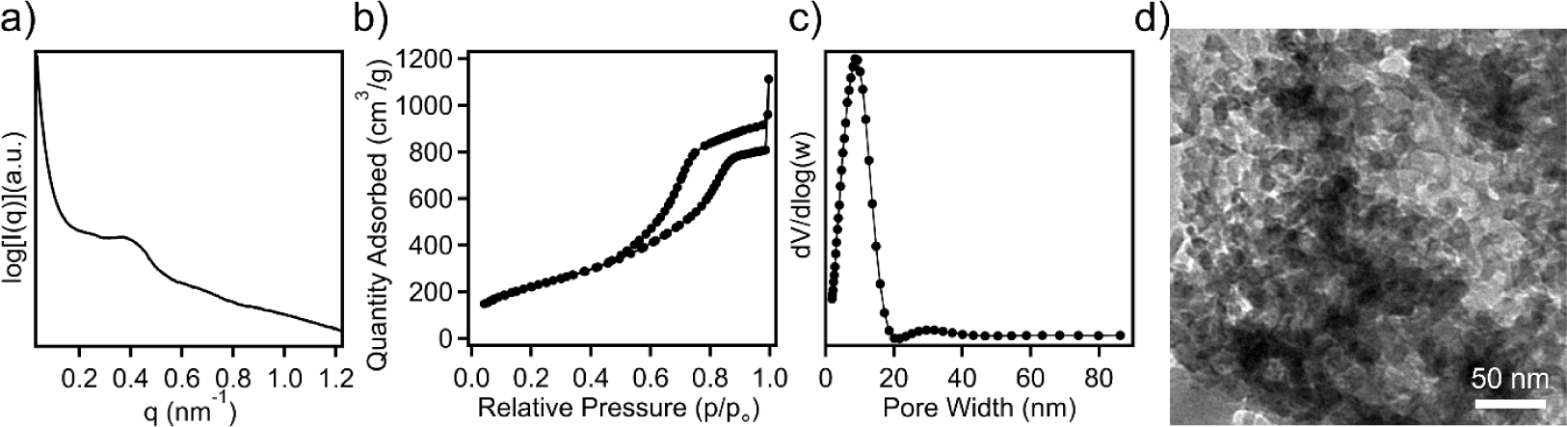
(a) SAXS profiles. (b) Nitrogen sorption isotherm, (c) pore size distribution, and (d) TEM micrograph of the OMS prepared using the SEBS89-derived OMC template (which was immersed in TEOS for 8 h).

Retention of ordered nanostructures following template removal can be confirmed through SAXS patterns presented in Figures S7 and 5a, where all samples exhibit a similar domain spacing of 16.3 nm. This domain spacing reduction following decomposition of the template is consistent with previous reports for hard-template synthesis of the OMS, where the framework undergoes thermal-induced shrinkage during template removal. Pore textures were further assessed through nitrogen physisorption in Figures S8 and 5b to determine how porosity evolves with varying soaking time. The surface area of the OMS was found to increase with a longer soaking time, from 572 m^2^/g with 1 h of reaction up to 902 m^2^/g with 8 h of reaction (Figure S9). Likewise, the pore volume exhibited a similar trend, increasing from 1.01 cm^3^/g to 1.58 cm^3^/g for the OMS prepared from 1 and 8 h reaction times. Figure 5c shows the pore size distribution of the 8 h sample centered at 8.6 nm, which has minimal variation with a reduced TEOS soaking time (Figure S10). The significant increase in surface area and pore volume in the OMS compared to both the carbon template and carbon–silica composite demonstrates the consistency of this TPE-derived OMC templating strategy. For comparison, large-pore OMS prepared with a surfactant/swelling agent pair were found to have a pore size of 23.1 nm and a specific surface area of 343 m^2^/g,^16^ whereas OMS derived from poly(ethylene oxide)-*b*-polystyrene led to a pore size of 30.8 nm and a surface area of 362 m^2^/g.^32^ In addition, the TEM micrograph in Figure 5d confirms the successful formation of an OMS using TPE-derived OMC templates.

**Figure 6:**
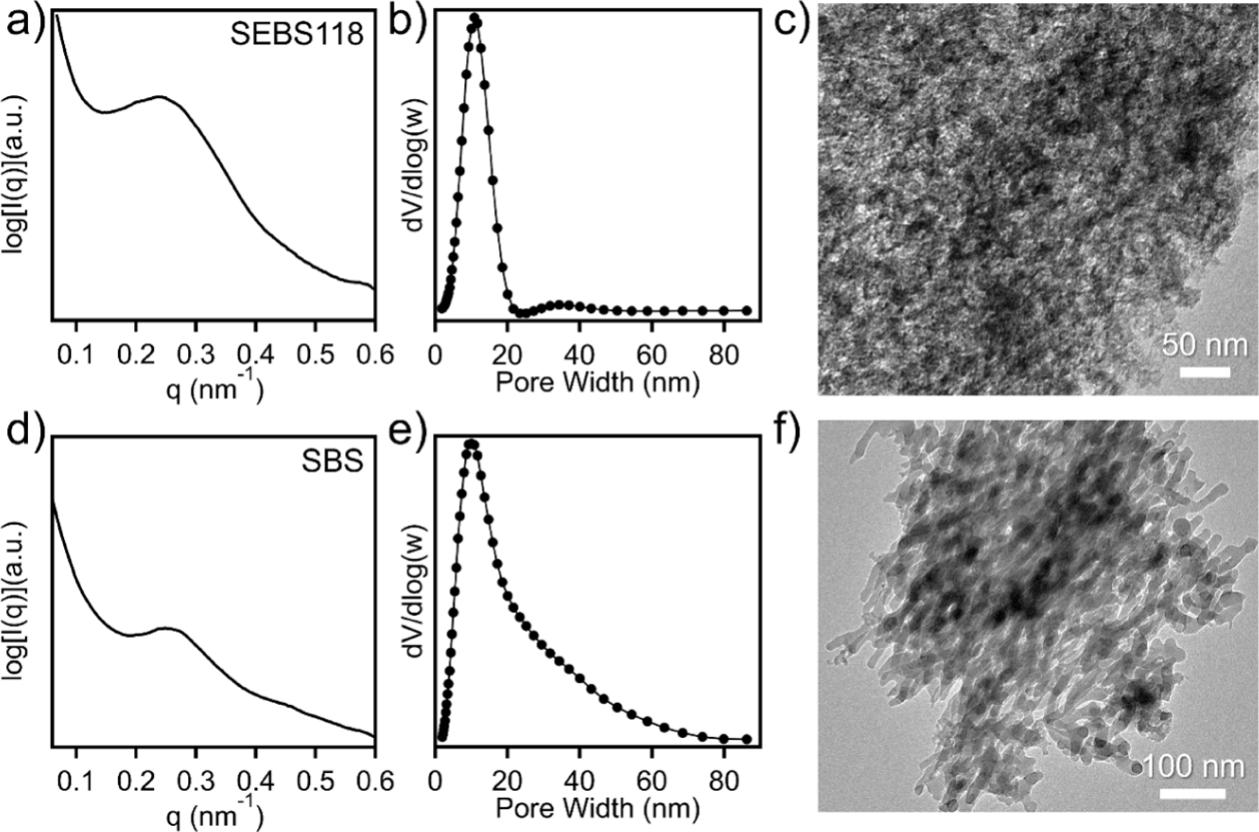
(a) SAXS profile, (b) pore size distribution, (c) and TEM micrograph of OMS templated by the SEBS118-derived OMC. (d) SAXS profile, (e) pore size distribution, (f) and TEM micrograph of OMS templated by the SBS-derived OMC.

The generalizability of this OMS fabrication strategy was then studied by extending it to different TPE precursors, which would enable tailorable pore characteristics dependent on initial TPE identity. First, an alternative commercial SEBS precursor (SEBS118) was utilized. In comparison to the model system, SEBS118 has a higher molecular weight (118 000 g/mol) with a similar PS volume fraction to SEBS89. Following the synthetic protocol consistent with the model system and decomposition of the template, a relative silica content of 23.5 wt % was observed (Figure S11). The higher molecular weight SEBS118 was found to result in a similar domain spacing in the resulting OMC (29.8 nm), as depicted in Figure S12. In turn, the OMS derived from the SEBS118-derived carbon template exhibited a domain spacing of 24.8 nm (Figure 6a). Moreover, the nitrogen physisorption isotherm and pore size distribution for the SEBS118-derived OMS are shown in Figures S13 and 6b with a pore size centered at 10.8 nm, a surface area of 703 m^2^/g, and a pore volume of 1.96 cm^3^/g. Figure 6c shows the presence of ordered mesopores of the SEBS118-derived OMS through TEM imaging. To further confirm the versatility of the OMS fabrication process, a second TPE was selected, which is a more efficient OMC precursor. The unsaturated polyolefin backbones in SBS can allow for an order of magnitude faster reaction kinetics compared to SEBS.^33^ Specifically, an SBS with a molecular weight of 140 000 g/mol and PS volume fraction of 0.33 was converted to the OMC templates. Figure S14 shows how the nanostructure evolves during cross-linking and pyrolysis, with a final OMC domain spacing of 31.6 nm. Following TEOS infiltration and carbon template removal, a relative silica content of 12.2 wt % was observed (Figure S15). The SBS-derived OMS exhibited a domain spacing of 23.8 nm, as depicted in Figure 6d, with a surface area of 850 m^2^/g and a pore volume of 2.23 cm^3^/g (Figure S16). The derived OMS has a pore size distribution centered at 10.0 nm, as shown in Figure 6e, though a broad shoulder toward larger pore sizes is observed. The porous structure of the SBS-derived OMC can be further probed through TEM imaging, where the mesoporous morphology can be observed. These results indicate that precursor selection can enable the controlled synthesis of OMS with varied mesopore sizes.

**Figure 7:**
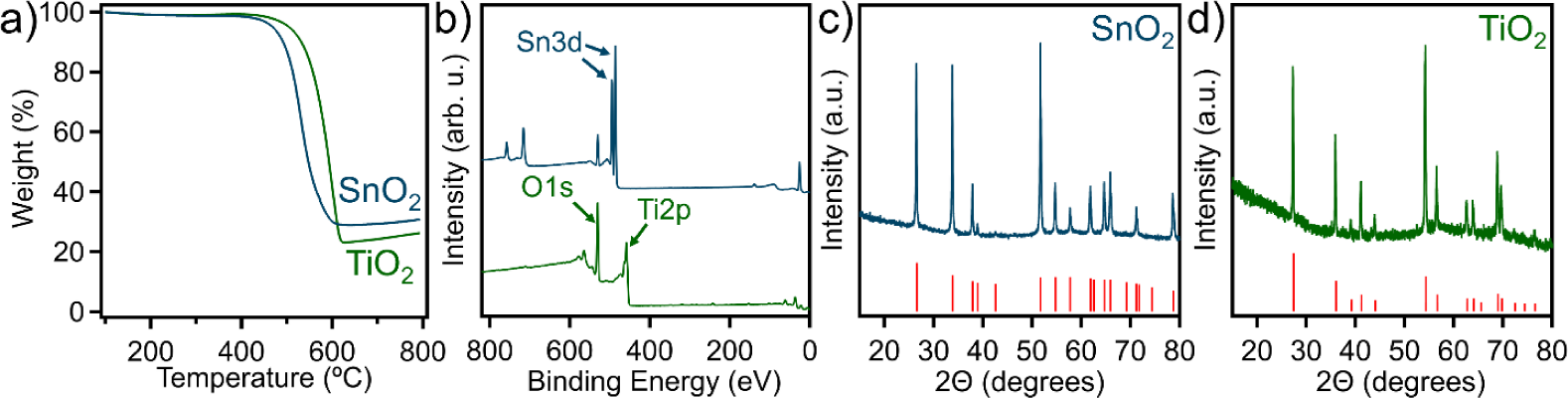
(a) TGA thermograms up to 800 °C under air and (b) XPS survey scans of OMTiO_2_ and OMSnO_2_. Representative XRD diffraction patterns of (c) aqueous OMSnO_2_ and (d) aqueous OMTiO_2_.

Furthermore, ordered mesoporous metal oxides (OMMOs) are a promising class of materials that have tailorable properties depending on the metal identity that allow for excellent performance in photocatalysis,^34,35^ energy devices,^36,37^ and sensors.^38^ For example, modulating surface functionality in porous materials has been shown to dramatically alter performance and expand their application space.^39−41^ Similar to OMS manufacturing, conventional OMMO synthetic strategies rely on the use of various templating agents, ranging from cationic or anionic surfactants such as alkyltrimethylammonium, ligands such as tetradecyl-phosphate, poly(alkylene oxide) block copolymers, and hard templates such as OMS.^39^ For example, the sol–gel approach involves the gradual polymerization of a precursor into a gel network, though this process may take days to weeks to complete. Both soft- and hard-templating have also been utilized to prepare OMMOs, though the same challenges (i.e., limited pore size from commercial surfactant templates) are typically observed as previously discussed with OMS synthesis. Through a similar protocol as described for OMS synthesis with SEBS89-derived OMC template, ordered mesoporous titanium oxide (OMTiO_2_) was prepared using titanium chloride as the precursor and ordered mesoporous tin oxide (OMSnO_2_) from tin chloride. Figure S17a,b shows the nitrogen physisorption isotherm and pore size distribution of the TPE-derived porous materials before and after loading with tin and titanium, where a reduction in the isotherms and pore size distributions is observed for both metal species. Moreover, the surface area decreased from 442 to 337 m^2^/g for the titanium composite and 391 m^2^/g for the tin composite, while the pore volume also decreased from 0.47 cm^3^/g to 0.34 cm^3^/g and 0.41 cm^3^/g for the titanium and tin composites, respectively. Following carbon template removal, a relative metal content of 30.8 and 26.2 wt % was found for the tin and titanium nanocomposites, respectively (Figure 7a). Additionally, the elemental composition can be determined from XPS survey scans shown in Figure 7b, where the tin chloride-derived sample exhibited ∼63.7 at. % oxygen and 36.2 at. % tin content. The binding environments were then studied through high-resolution XPS scans in Figure S18, where tin has two peaks present at 494.6 eV corresponding to Sn 3*d*_3/2_ and at 486.2 corresponding to Sn 3*d*_3/2_. In addition, only one O 1*s* peak is present at 530.1 eV. Similarly, the titanium chloride-derived sample had 65.3 at% oxygen and 32.3 at% titanium, showing both metal oxides are approximately at a 1:2 atomic ratio of metal to oxygen. Moreover, a high-resolution XPS scan for Ti 2*p* shows four deconvoluted peaks associated with TiO_2_, which are at 459.8 eV corresponding to Ti 2*p*_3/2_, 463.8 eV corresponding to Ti 2*p*_1/2_, 457.9 eV associated with Ti(III) oxide, and 471.2 eV (a satellite peak, Figure S19), respectively. Additionally, O 1*s* exhibited two peaks at 529.8 and 530.8 eV associated with Ti–O bond. In order to understand the crystalline structure of these metal oxides, Figure 7c,d presents representative XRD patterns for both OMSnO_2_ and OMTiO_2_, respectively, where both structures display high crystallinity. For OMSnO_2_, the diffraction peaks at 26.4°, 33.7°, 37.9°, 51.7°, 54.7°, 61.8°, 64.7°, 65.9°, and 71.2° corresponding to (110), (001), (200), (211), (220), (310), (112), (301), and (202) reflections of cassiterite-type crystalline walls (JCPDS 41–1445).^42^ Additionally, the OMTiO_2_ diffraction peaks at 27.3°, 35.9°, 39.0°, 41.1°, 43.9°, 54.2°, 56.5°, 62.6°, 63.8°, 68.8°, and 69.6° corresponding to (110), (101), (200), (111), (210), (211), (220), (002), (310), (301), and (112) reflections of rutile TiO_2_ (JCPDS 21–1276).^43^ These results indicate the pore walls consist of nanocrystalline metal oxide particles in both systems, which has been observed in several reports for both mesoporous tin and titanium oxide.^44−47^ It should be noted that the carbon template contains ∼0.7 at% sulfur (Figure 4b), which may promote OMMO fabrication through efficient precursor infiltration due to sulfur’s ability to coordinate with metal species.^48,49^

**Figure 8:**
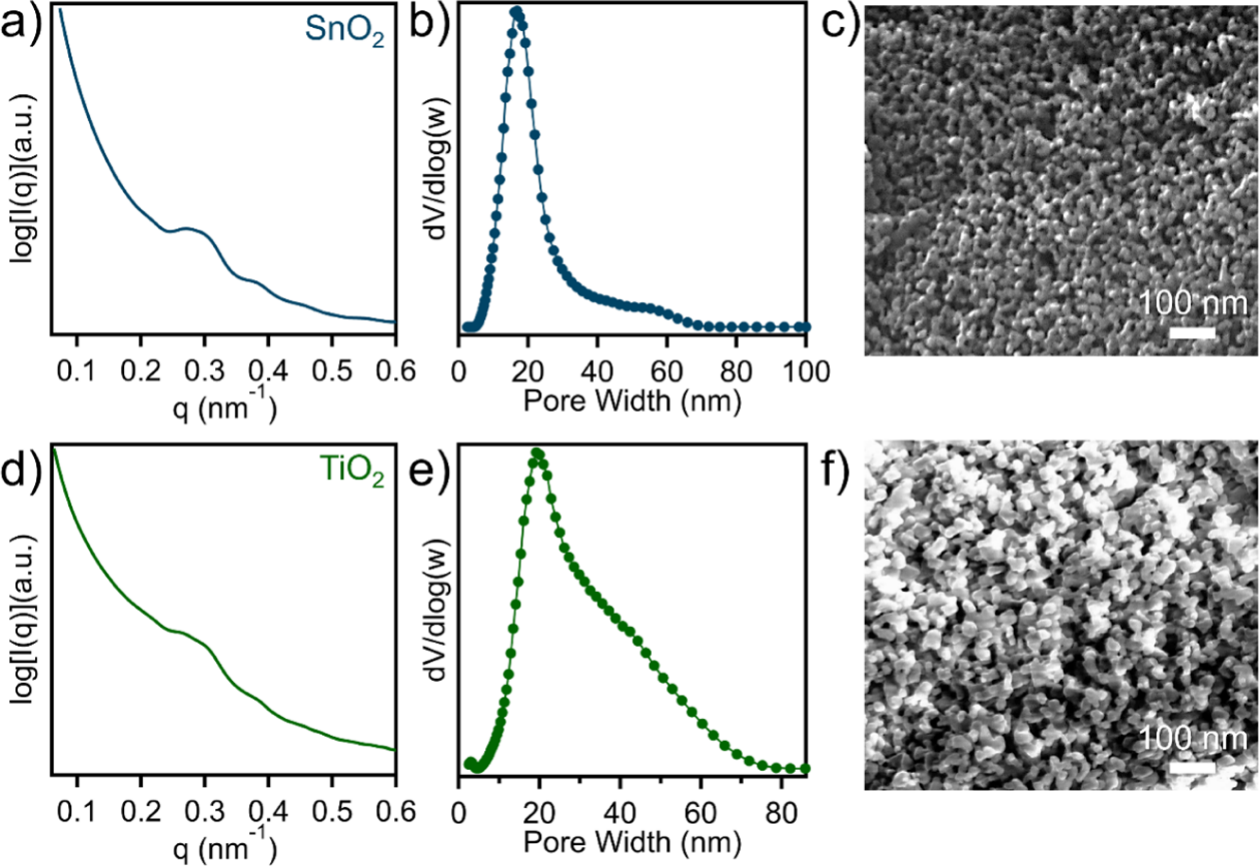
(a) SAXS profile, (b) pore size distribution, and (c) SEM micrograph of OMSnO_2_. (d) SAXS profile, (e) pore size distribution, (f) and SEM micrograph of OMTiO_2_.

The nanostructure of the tin oxide was then examined through SAXS, as shown in Figure 8a, where a primary ordering peak was observed with a domain spacing of 21.6 nm. Furthermore, a broad secondary ordering peak was also observed, which suggests a high degree of ordering, similar to the OMS at low reaction times. Nitrogen physisorption experiments in Figures 8b and S20 show the tin oxide exhibited a pore size of 16.9 nm, a relatively low surface area of 72 m^2^/g, and a pore volume of 0.470 cm^3^/g. These surface areas and pore volumes are similar to OMSnO_2_ fabricated from several synthetic strategies,^44,45,50^ while the relatively large pore size demonstrates the advantages of this synthetic route. Pore formation within the tin matrix was then further confirmed through SEM imaging in Figure 8c, where the OMSnO_2_ was found to have an average pore size of 15.5 nm. In addition, the titanium oxide nanostructure was also investigated through SAXS (Figure 8d), where a primary ordering peak was observed with a corresponding domain spacing of 21.9 nm. The similar nanostructure size suggests this TPE-derived OMMO strategy is generalized and consistent, while the resulting pore texture is mostly influenced by the identity of TPE templates. The titanium oxide had a pore size of 19.2 nm, a surface area of 92 m^2^/g, and a pore volume of 0.537 cm^3^/g (Figures 8e and S21). The porous structure of OMTiO_2_ was also characterized through SEM imaging, as shown in Figure 8f, which further supports the successful formation of uniform pore sizes (average pore size: 18.5 nm). Through the extension of this synthetic method to different metal oxide precursors, the versatility of this strategy in preparing functional nanomaterials with varied functionalities is demonstrated.

## 4 Conclusions

In this work, we demonstrate the use of commodity thermoplastic elastomers (TPEs) as structure-directing agents for OMM synthesis, including ordered mesoporous silica (OMS) and metal oxides (OMMOs). Specifically, TPEs, such as poly(styrene)-*block*-poly(ethylene-*ran*-butylene)-*block*-poly(styrene) and poly(styrene)-*block*-poly(butadiene)-*block*-poly(styrene), were directly converted to ordered mesoporous carbon and utilized as replicas for OMS and OMMO production. The impact of introducing inorganic precursors into the carbon template on the nanostructure of derived porous materials was systematically investigated by utilizing TEOS infiltration as a model system. Following template decomposition, an OMS with relatively large pores (8.6 nm) can be prepared. It was also found that the pore texture of the OMS, including surface area and porosity, can be modulated by varying the starting TPE’s chemical composition. Furthermore, we demonstrate the fabrication of large-pore OMMOs using TPE-derived carbon templates with tin and titanium precursors. Overall, this work provides a robust OMM manufacturing strategy that can achieve tailorable pore textures and matrix chemistries with scalable processes and low-cost precursors.

## Data Availability

The data are available from the corresponding author upon reasonable request.

## Supporting Information Available

Nitrogen sorption isotherms, pore size distributions, pore volumes/surface areas, and TGA thermograms for carbon–silica composites prepared from various infiltration times. High-resolution XPS scans for the SEBS89-derived OMC template, carbon–silica composite prepared from an 8-h infiltration time, and corresponding OMS. SAXS profiles, nitrogen sorption isotherms, pore size distributions, and pore volumes/surface areas for OMS prepared from various infiltration times. TGA thermograms, SAXS profiles, nitrogen sorption isotherms, for SEBS118- and SBS-derived OMS. Nitrogen sorption isotherms and pore size distributions for tin- and titanium-loaded SEBS89-derived OMC. High-resolution XPS scans and nitrogen sorption isotherms for OMSnO_2_ and OMTiO_2_ (PDF)
