# Supplementary material for: Commodity Thermoplastic Elastomer-Enabled Templated Synthesis of Large-Pore Ordered Mesoporous Materials
Source: ACS Omega. 2025 Mar 12;10(11):11554–61. doi: 10.1021/acsomega.5c00553 (PMC11947817; doi:10.1021/acsomega.5c00553)
Supplement: Supplementary file 1 — ao5c00553_si_001.pdf [file ao5c00553_si_001.pdf]

Electronic Supplementary Information for:

Commodity thermoplastic elastomer-enabled templated synthesis of large pore ordered mesoporous materials

Anthony Griffin,[a] Parker Frame,[a] Yizhi Xiang,[b] Zhe Qiang[a,*]

[a]School of Polymer Science and Engineering, University of Southern Mississippi, Hattiesburg, MS, 39406, USA

[b]Department of Chemical and Biomedical Engineering, University of Missouri, Columbia, MO, 65211, USA

Corresponding authors: Z. Q. (zhe.qiang@usm.edu)

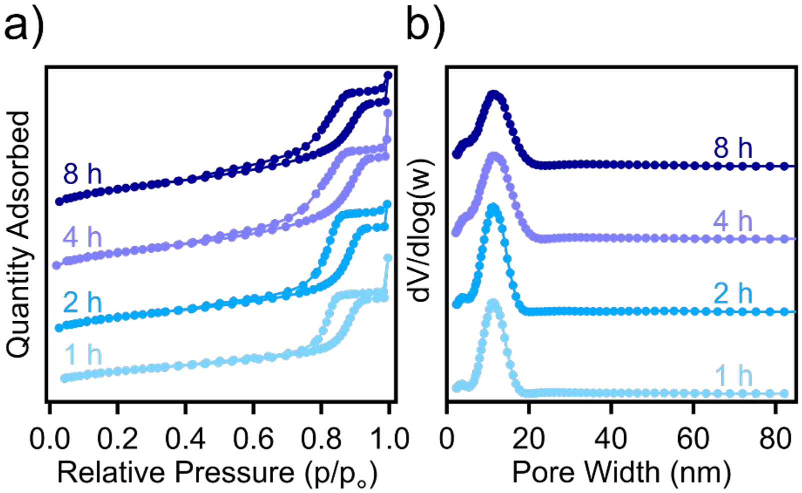

Figure S1. (a) Nitrogen sorption isotherms and (b) corresponding pore size distributions as a function of reaction time for carbon-silica composites following pyrolysis.

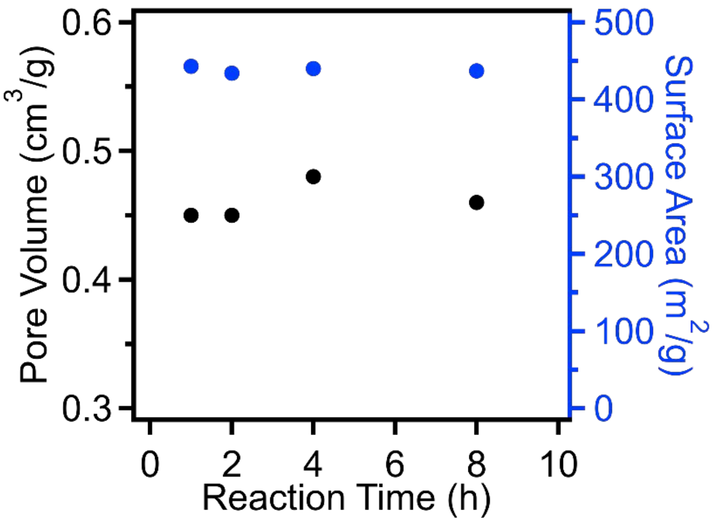

Figure S2. Pore volume and surface area as a function of reaction time for carbon-silica composites following pyrolysis.

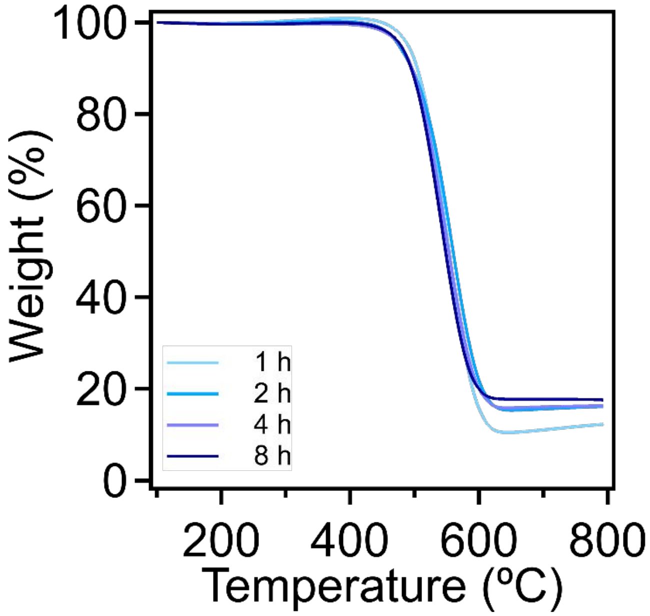

Figure S3. TGA thermograms up to 800 °C under air for carbon-silica composites with varied infiltration times.

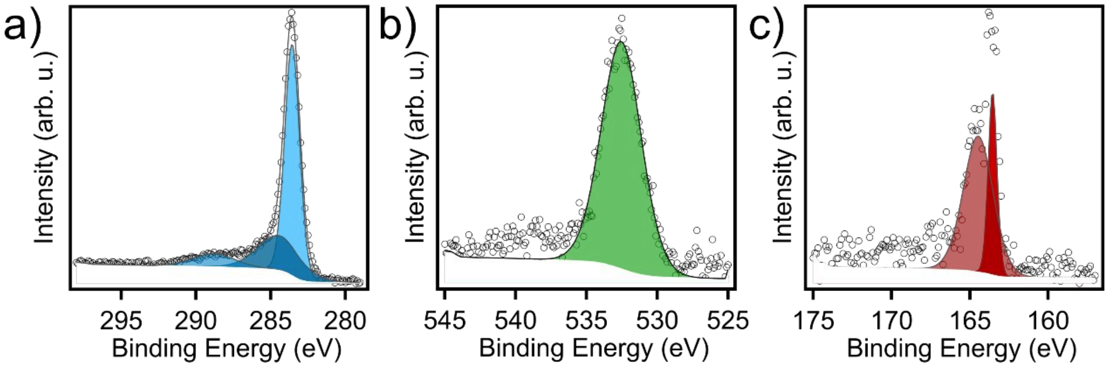

Figure S4. High-resolution XPS scans depicting (a) carbon, (b) oxygen, and (c) sulfur bonding environments for the SEBS89-derived OMC template.

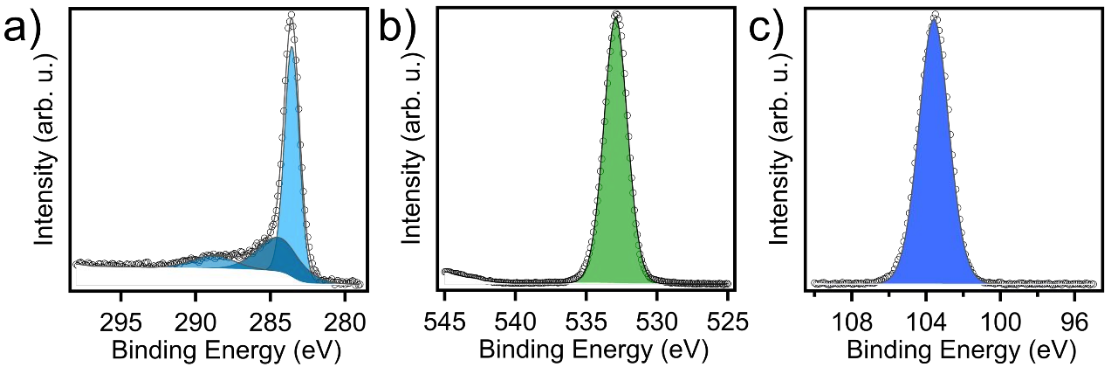

Figure S5. High-resolution XPS scans depicting (a) carbon, (b) oxygen, and (c) silica bonding environments for a carbon-silica composite reacted for 8 h.

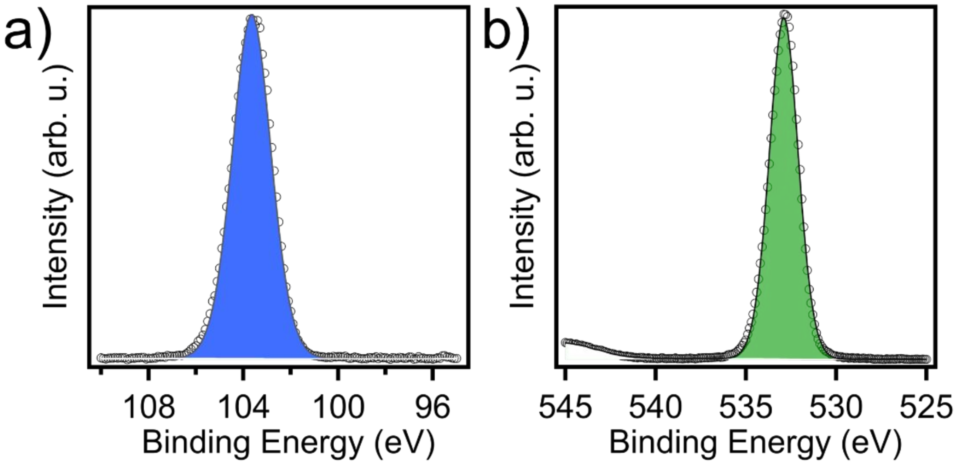

Figure S6. High-resolution XPS scans depicting (a) silica and (b) oxygen bonding environments for SEBS89-derived OMS reacted for 8 h.

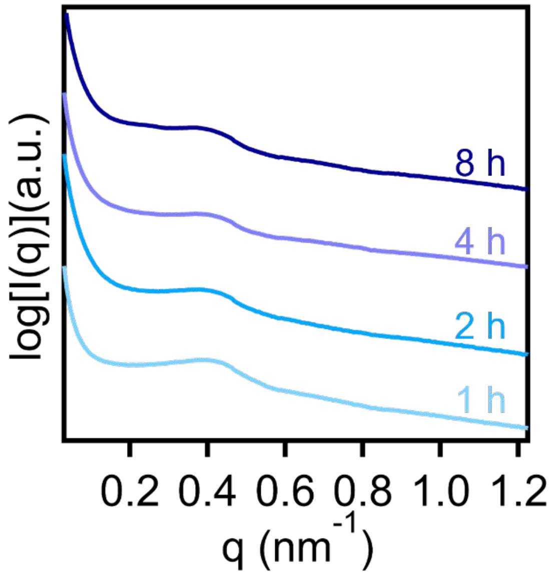

Figure S7. SAXS profiles for SEBS89-derived OMS with varied infiltration times.

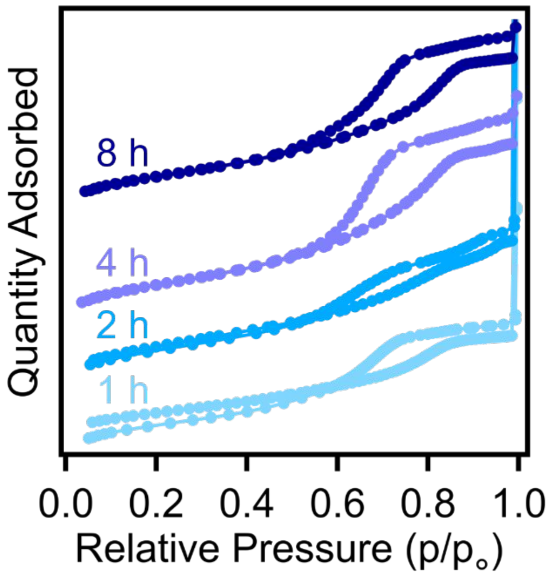

Figure S8. Nitrogen sorption isotherm for SEBS89-derived OMS with varied infiltration times.

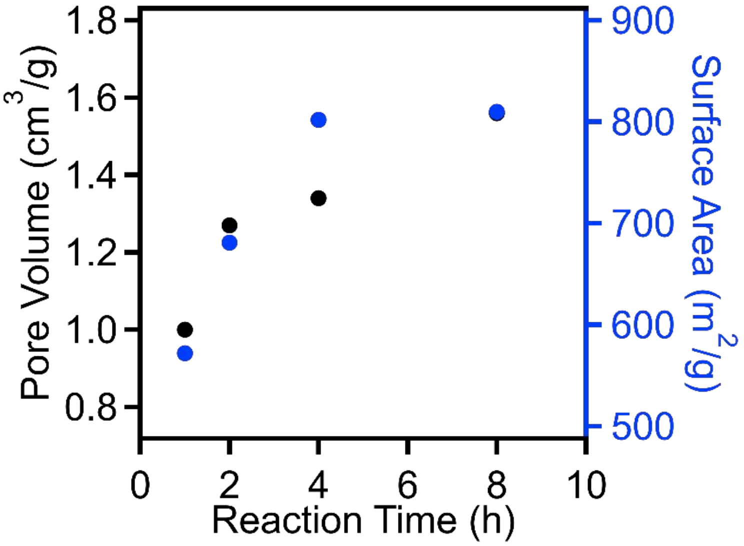

Figure S9. Pore volume and surface area as a function of reaction time for OMS.

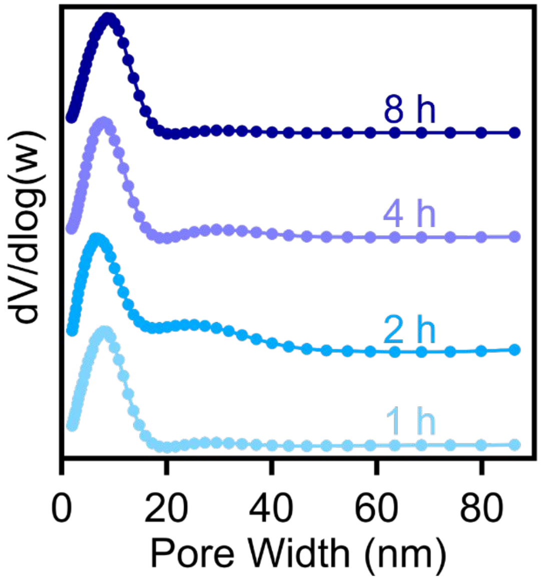

Figure S10. Pore size distributions for SEBS89-derived OMS with varied infiltration times.

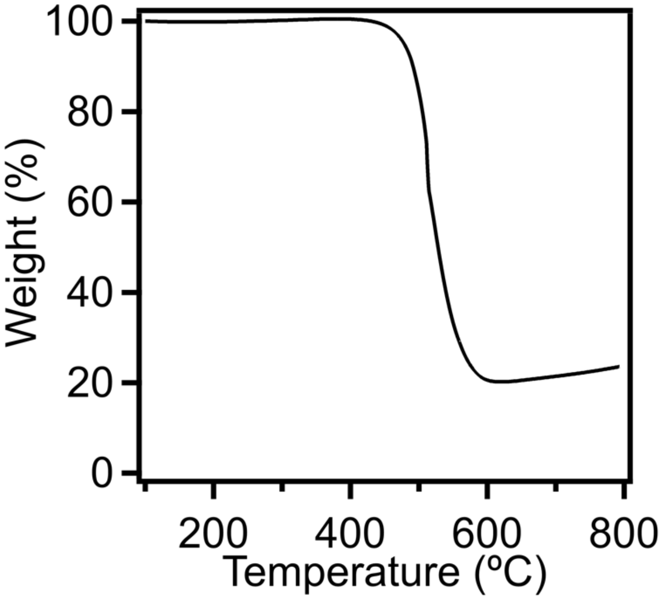

Figure S11. TGA thermograms up to 800 °C under air for SEBS118-derived OMS.

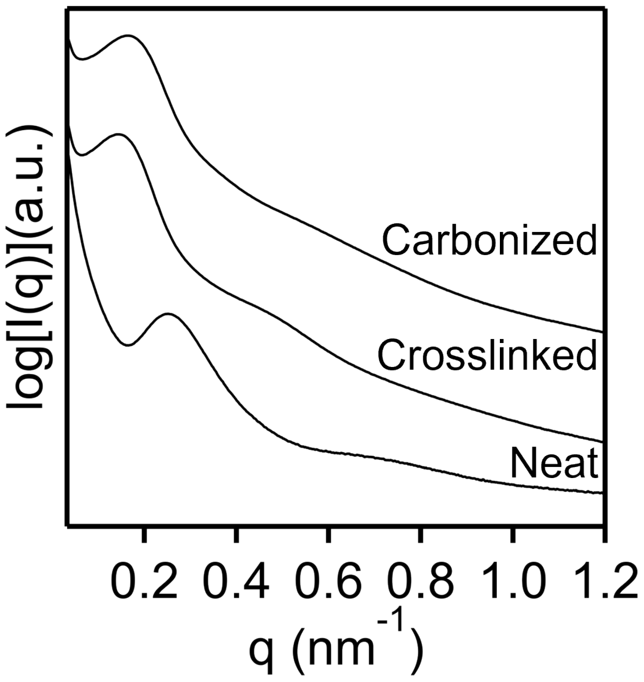

Figure S12. SAXS profiles for neat, crosslinked, and carbonized SEBS118.

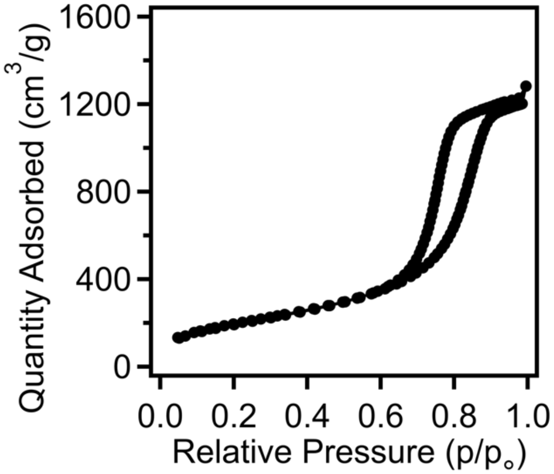

Figure S13. Nitrogen sorption isotherm for SEBS118-derived OMS.

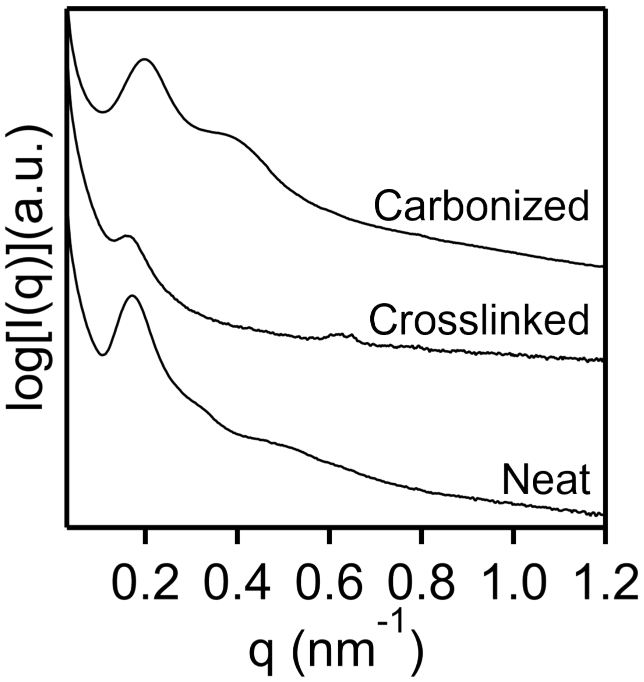

Figure S14. SAXS profiles for neat, crosslinked, and carbonized SBS.

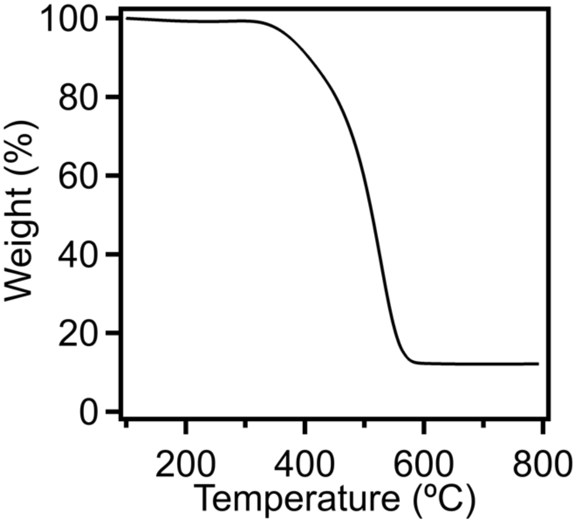

Figure S15. TGA thermograms up to 800 °C under air for SBS-derived OMS.

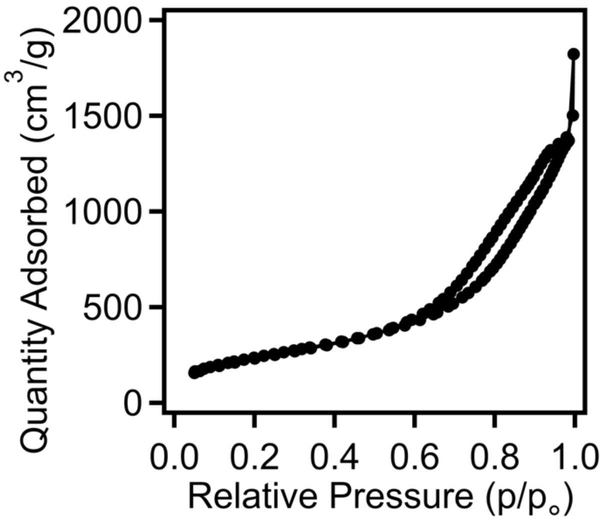

Figure S16. Nitrogen sorption isotherm for SBS-derived OMS.

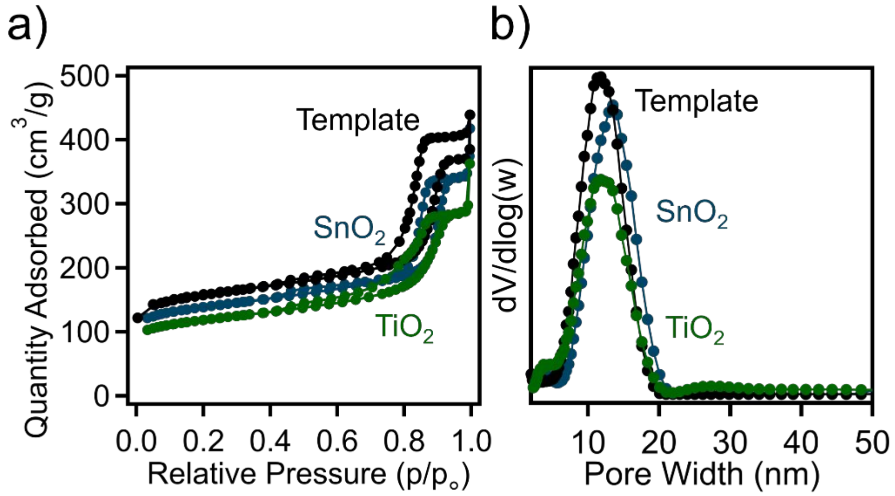

Figure S17. (a) Nitrogen sorption isotherms and (b) pore size distributions for SEBS89-derived OMC before and after loading with tin and titanium.

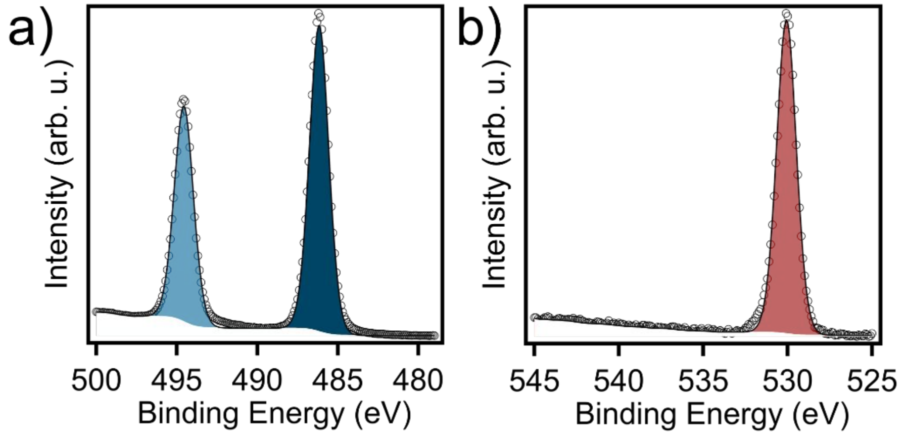

Figure S18. High-resolution XPS scans depicting (a) tin and (b) oxygen bonding environments for $OMSnO_2$.

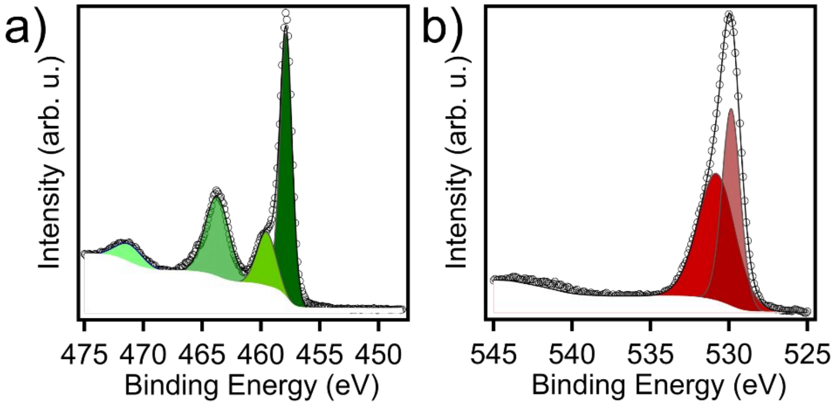

Figure S19. High-resolution XPS scans depicting (a) titanium and (b) oxygen bonding environments for $OMTiO_2$.

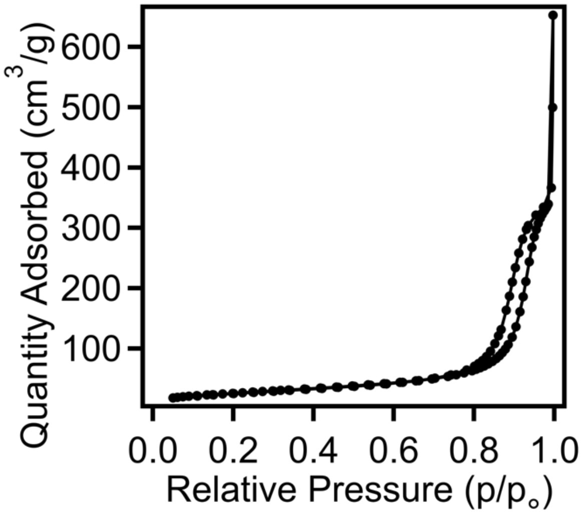

Figure S20. Nitrogen sorption isotherm for $OMSnO_2$.

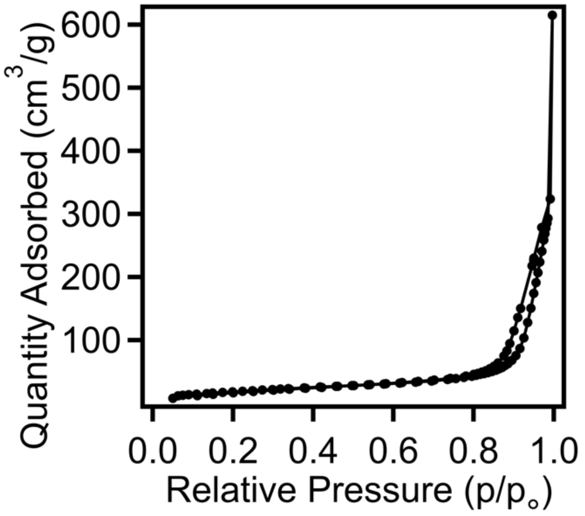

Figure S21. Nitrogen sorption isotherm for $OMTiO_2$.
